# Epidemiology of in-hospital cardiac arrest in a Pakistani tertiary care hospital pre- and during COVID-19 pandemic

**DOI:** 10.12669/pjms.38.ICON-2022.5776

**Published:** 2022-01

**Authors:** Faiza Ahmed, Lubna Abbasi, Nida Ghouri, Muhammad Junaid Patel

**Affiliations:** 1 Dr. Faiza Ahmed, (FCPS II Trainee Year 4, Internal Medicine), Training R4 Internal Medicine, Department of Internal Medicine, Indus Hospital and Health Network Karachi, Pakistan; 2 Dr. Lubna Abbasi, FCPS. Internal Medicine/Rheumatology, Department of Internal Medicine, Indus Hospital and Health Network Karachi, Pakistan; 3 Nida Ghouri, MS Microbiology. Research Associate, Indus Hospital Research Center, Indus Hospital & Health Network, Karachi, Pakistan; 4 Dr. Muhammad Junaid Patel, (Diplomat American Board of Internal Medicine), Internal Medicine, Department of Internal Medicine, Indus Hospital and Health Network Karachi, Pakistan

**Keywords:** In-hospital cardiac arrest, COVID-19, Gender, Causes of Cardiac arrest

## Abstract

**Objectives:**

To determine epidemiology of in-hospital cardiac arrest (IHCA) in a tertiary care hospital, pre- and during pandemic.

**Methods:**

This is a cross-sectional study of inpatients who experienced an in-hospital-cardiac arrest at a tertiary care hospital in Karachi between August 2019 and August 2020. Outcome variables were return of spontaneous circulation (ROSC) and survival to discharge (StD) and analysis was also done comparing pre- and during pandemic period.

**Results:**

A total of 77 patients experienced at least one IHCA event during the 1-year study period. Comparing pre- and during pandemic, ROSC for women was higher during the pandemic albeit not significant (43% vs 50%) in comparison to men (54% vs 10%, p<0.001). During the pandemic, women with IHCA were significantly younger than men (μ ± sd; 36.8 ± 15.3 vs 55.9 ± 12.7, p=0.001,) whereas pre-pandemic, there was no gender differences in mean age. Non-shockable rhythm was more common (92.2%) than shockable rhythm (6.5%). Pre- and during pandemic, there were significant differences in the cause of IHCA for 4H4T (87% vs 100%) and cardiac (36% vs 9%). The proportion of hypoxic patients increased from 50% during pre-pandemic to 91% during the pandemic period, whereas hypo/hyperkalemia decreased from 53% to 34%.

**Conclusion:**

Despite the limitation of a small sample size, our study has provided important information regarding the epidemiology and outcomes of IHCA pre- and during pandemic in a busy Pakistani tertiary care hospital. Our finding that gender differences exist in survival pre- and during pandemic needs to be explored further with more hospitals doing comparative studies.

## INTRODUCTION

In-hospital cardiac arrest (IHCA) is a major cause of death worldwide yet, data on the epidemiology of IHCA is limited.^1^ Cardiac-related causes such as heart failure, arrhythmia or myocardial infarction account for the majority of the cases (50%-60%) followed by respiratory insufficiency as a leading cause.^2^ Survival outcomes after an IHCA event vary between 0% to 42% globally.^3^ Some major patient-related factors age, gender, initial presentation, underlying conditions; whereas major healthcare related factors include response time of emergency team, location of event, duration and method of resuscitation.^1^,^4^-^7^ Studies have reported that patients with a shockable rhythm have upto 2 to 3 times higher survival to hospital discharge (StD) in comparison to patients with a nonshockable rhythm.^8^,^9^

The recent COVID-19 pandemic is further impacting the epidemiology and outcome with some studies indicating an increase in the burden of cardiac arrest in COVID-19 patients.^3^ A US-based study showed that during the first peak of the pandemic, there was an almost five-fold increase in incidence of IHCA as compared to the same period in the previous year.^10^ In the first meta-analysis comparing outcomes in patients with IHCA before and during the COVID-19 pandemic, concluded that even though cardiac arrests in COVID-19 patients was higher, the return of spontaneous circulation (ROSC) was similar in the pre- and COVID-19 periods as was overall mortality.^11^

There is evidence elsewhere that recognizing the cause of arrest by the emergency team as well as minimizing health-care related risk factors may help in improving survival outcomes.^2^ With limited published literature from Pakistan on the epidemiology of IHCA in adults and evidence that the COVID-19 pandemic is associated with a higher incidence of arrests with worse survival rates, in the present study, we aimed to investigate the profile and outcomes of IHCA patients pre- and during the COVID-19 pandemic.

## METHODS

A cross-sectional study was conducted on patients who experienced an IHCA event at the Korangi Campus of the Indus Hospital and Health Network between Aug 2019 and Aug 2020. It included patients admitted in Emergency (ER), wards, intensive care unit (ICU), critical care unit (CCU), high dependency unit (HDU) and COVID-19 ICU. Only the initial cardiac arrest was taken if any patient had more than one.

A pre-coded questionnaire was used to capture data. Sociodemographic variables included were age, gender, and comorbidities whereas IHCA event variables were initial cardiac rhythm, Hs & Ts, event location, pre-arrest cerebral performance and any underlying factors present prior to arrest. Pre-pandemic period was defined as August 2019 to Feb 2020 and during-pandemic was from March 2020 to August 2020. An IHCA event was defined as a cardiac arrest that occurs in a hospital and for which resuscitation was attempted with chest compressions, defibrillation, or both. Outcome variables were return of spontaneous circulation (ROSC) and survival to discharge (StD).

All patients included in the study were age 14 and above. In our hospital, 14-17 years are treated as adults. Patients excluded were those below 14 years, those who had an out-of-hospital-cardiac-arrest, were transferred in-patients from another hospital with a history of arrest prior to arrival to our hospital, patients on Temporary Pacemaker (TPM) or Permanent Pacemaker (PPM) and those post cardiac catheterization. Approval from Institution Review Board was taken (IRD_IRB_2019_06_006).

Data was entered and analyzed using software SPSS 26. Mean ± SD was calculated for age based on normality. Frequency and percentage were calculated for all the categorical variables. Chi-square or Fischer exact test was applied to see the association of all the categorical variables with ROSC, StD and period of cardiac arrest.

## RESULTS

**Fig.1:**
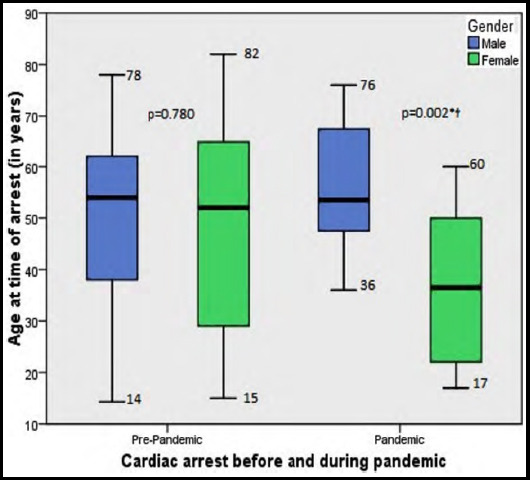
Association of age and gender with IHCA.

A total of 77 patients (57% men) experienced at least one IHCA event during the 1-year study period Table-I. Overall, 39% IHCA patients achieved return to spontaneous circulation (ROSC) and 36% survived to discharge (StD) from the hospital. Majority of the cardiac arrest for those who survived was in the Emergency department (57%) followed by ICU (13%). ROSC and StD was significantly higher in non-ICU patients as compared to ICU patients (p<0.0001, Table-I. It was also observed that women who underwent cardiac arrest during the pandemic were significantly younger than men (μ ± sd; 36.8 ± 15.3 vs 55.9 ± 12.7, p=0.001) whereas before the pandemic, there was no differences in their mean age (Fig.1). Non-shockable rhythm was more common (92.2%) in these patients than shockable rhythm (6.5%).

**Table I:** Characteristics and outcomes of patients who experienced an IHCA in a tertiary-care hospital in Karachi Pakistan, 2019-2020.

Variables	All	ROSC	p-value	Survival at Discharge	p-value
	
	n (%)			n(%)	
All IHCA patients, n (%)	77	30 (39)	-	28 (36.4)	-
**Gender; n(%)**					
Male	44 (57.1)	15 (50)	0.312^□^	14 (50)	NS
Female	33 (42.9)	15 (50)		14 (50)	
**Location of cardiac arrest; n(%)**					
Emergency department (ED)	20 (26.0)	17 (56.7)	0.000^**‡^	17 (60.7)	0.000^**‡^
Intensive care unit (ICU)	21 (27.3)	4 (13.3)		4 (14.3)	
COVID ICU	23 (29.9)	3 (10)		1 (3.6)	
Other (ward, HDU, etc)	13 (16.9)	6 (20)		6 (21.4)	
**Initial cardiac rhythm; n(%)**					
Shockable rhythm					
Ventricular fibrillation	4 (5.2)	2 (6.9)	NS	2 (7.4)	NS
Pulseless ventricular tachycardia	1 (1.3)	-		-	
Non-shockable rhythm					
Pulseless electrical activity	10 (13)	1 (3.4)		1 (3.7)	
Asystole	61 (79.2)	26 (89.7)		24 (88.9)	
**Pre arrest cerebral performance; n(%)**					
Mild to no neurological disability	51 (66.2)	22 (75.9)	NS	20 (74.1)	NS
Moderate disability	7 (9.1)	1 (3.4)		1 (3.7)	
Severe disability	5 (6.5)	2 (6.9)		2 (7.4)	
Coma or vegetative state	12 (15.6)	4 (13.8)		4 (14.8)	
Brain death	1 (1.3)	4 (13.8)		-	
**Factors present prior to Arrest; n(%)**					
Mechanical ventilation	31 (44.9)	4 (15.4)	0.005^*□^	3 (12)	0.001^*□^
Renal insufficiency	36 (52.2)	11 (42.3)		10 (40)	
Hepatic insufficiency	10 (14.5)	4 (15.4)		4 (16)	
Sepsis	34 (49.3)	12 (46.2)		12 (48.0)	
Malignant disease	2 (2.9)	-		-	
Hypotension	29 (42.0)	9 (34.6)		8 (32.0)	

* p<0.05,

** p<0.0001, NS not significant,

□ Chi square test,

‡ Fisher exact test.

Approximately 58% of the IHCA events occurred pre-pandemic. Pre-pandemic for both ROSC as well as StD nearly half of our patients recovered. However, ROSC dropped to 25% and StD to 18.8% during the pandemic. Even though not statistically significant, there was an increase in the proportion of 30-39 year old age group experiencing an IHCA for men and women. There were also differences in ROSC between men and women pre-and during pandemic. For men, there was statistically significant reduction in recovery from 54% in pre-pandemic to just 10% during pandemic. However, for women, there was no difference in recovery pre- and during pandemic (43% vs 50%) Table-II.

**Table II:** Causes of patients who experienced an IHCA in pre pandemic and during pandemic.

Variables, n (%)	Time of cardiac arrest			
	Pre-Pandemic (Aug ‘19 – Feb ’20) n=45	Pandemic (Mar-Aug 2020) n=32	Total n=77	p-value
**ROSC**	22 (48.9)	8 (25)	30 (39)	0.034*^□^
**Survive to discharge**	22 (48.9)	6 (18.8)	28 (36.4)	0.007^*□^
**Age (years)**				
**Male**				
<30 year	4 (16.7)	-	4 (9.1)	NS
30-39 year	2 (8.3)	3 (15)	5 (11.4)	
40-59 year	11 (45.8)	9 (45)	20 (45.5)	
60-69 year	4 (16.7)	3 (15)	7 (15.9)	
70 and above	3 (12.5)	5 (25)	8 (18.2)	
**Female**				
<30 year	6 (28.6)	4 (33.3)	10 (30.3)	NS
30-39 year	3 (14.3)	3 (25)	6 (18.2)	
40-59 year	3 (14.3)	4 (33.3)	7 (21.2)	
60-69 year	6 (28.6)	1 (8.3)	7 (21.2)	
70 and above	3 (14.3)	-	3 (9.1)	
**Gender and ROSC; n (%)**				
**Male**	n=24	n=20	n=44	
ROSC	13 (54.2)	2 (10)	15 (34.1)	0.002^*□^
**Female**	n=21	n=12	n=33	
ROSC	9 (42.9)	6 (50)	15 (45.5)	NS
**Gender and survival; n (%)**				
**Male**	n=24	n=20	n=44	
Survived to discharge	13 (54.2)	1 (5)	14 (31.8)	0.000^**□^
**Female**	n=21	n=12	n=33	
Survived to discharge	9 (42.9)	5 (41.7)	14 (42.4)	NS
**Initial cardiac rhythm**				
Shockable rhythm				
Ventricular fibrillation	3 (3.8)	1 (3.1)	4 (5.3)	
Pulseless ventricular tachycardia	1 (2.3)	-	1 (1.3)	
Non-shockable rhythm				NS
Pulseless electrical activity	8 (18.2)	2 (6.3)	10 (13.2)	
Asystole	32 (72.7)	29 (90.6)	61 (80.3)	
**Predominant cause of IHCA; n (%)**				
Cardiac	16 (35.6)	3 (9.4)	19 (24.7)	0.009^*‡^
4H4T	39 (86.7)	32 (100)	71 (92.2)	0.038^*□^
Sepsis	23 (51.1)	13 (40.6)	36 (46.8)	NS
**Breakdown of 4H4T; n (%)**				
Hypoxia	20 (50)	29 (90.6)**^a^**	49 (68.1)	0.006^*□^
Hypovolemia	1 (2.5)	-	1 (1.4)	
Hypo/Hyperkalemia	21(52.5)	11 (34.4)	32 (44.4)	
Thrombosis/pulmonary embolus	2 (5)	1 (3.1)	3 (4.2)	
Toxication	2 (5)	2 (6.3)	4 (5.6)	
Hydrogen ion (acidosis)	25 (62.5)	16 (50)	41 (56.9)	

* p<0.05,

** p<0.0001,

□ Chi square test,

‡ Fisher exact test,

ɫ Independent sample t test

Within non-shockable rhythm, PEA as a cause decreased from 18% to 6% pre- and during-pandemic, whereas unexpectedly, asystole as initial cardiac rhythm increased from 73% to almost 91% during pandemic. Pre- and during pandemic, there were significant differences in the cause of IHCA for cardiac (36% vs 9%, p=0.0009) and 4H4T (87% vs 100%, p=0.04) respectively. The proportion of patients with sepsis remained the same (51% vs 41%). Within 4H4T causes, the proportion of hypoxic patients increased from 50% during pre-pandemic to 91% during the pandemic period, whereas hypo/hyperkalemia decreased from 53% to 34% (p=0.006, Table-II).

## DISCUSSION

A few publications have started emerging regarding outcomes of in-hospital cardiac arrest during the COVID-19 pandemic yielding information regarding gender, age, common initial rhythm, survival to discharge, or achievement of ROSC.^3^,^10^-^14^ To the best of our knowledge, this is the first study reporting the epidemiology and outcomes of IHCA pre- and during pandemic in Pakistan.

The number of IHCA episodes that took place pre- and during pandemic at our hospital were comparable. As expected, the proportion of those who survived cardiac arrest reduced during the pandemic. Sandroni et al aptly summarized some reasons for the pandemic affecting the epidemiology of in-hospital cardiac arrest.^3^ The authors summarized that some possibilities of reduced survival could be due to restrictive guidelines for the management of cardiac arrest which may include extra donning and doffing of protective gear leading to delays in starting CPR as well as a large influx of severely ill patients which overwhelmed the hospital systems. Lim et al’s review study noted during the pandemic, in-hospital cardiac arrest incidence of COVID-19 patients varied between 1.5% and 5.8% among hospitalized patients and specifically between 8.0–11.4% among patients in ICU. In-hospital cardiac arrest occurred more commonly in older male patients while most common initial rhythms were non-shockable (83.9%, [asystole = 36.4% and pulseless electrical activity = 47.6%]).^13^ The proportion in our data also showed that during non-shockable was nearly 91%; however, the asystole was much higher (91%) as compared to PEA which was just 6%. Another study conducted during the COVID -19 pandemic showed asystole as the most common initial rhythm. It is odd to note this such that the expected rhythm should have been Pulseless electrical activity as it is the commonest result of respiratory failure and hypoxia^15^ as expected from COVID-19. Our sample size was too small and more work needs to be done to investigate if asystole is also as common in hypoxia and whether this is limited to COVID-19 related hypoxia alone.

Our finding was similar to other studies in terms that a larger proportion of men have a cardiac arrest.^16^ However, we found that during the pandemic, the proportion of men who did not achieve ROSC dropped significantly whereas that was not the case for women. We do not have an explanation for this finding. It is possible that men were more likely to have been exposed to COVID-19 as opposed to the women presenting in the Emergency. This hypothesis cannot be verified since it is not common practice to do a post-mortem or even a COVID-19 test on people who die in emergency in Pakistan. Our study shows survival to discharge and achievement of ROSC were both higher among non-ICU patients, similar to a previous study where return of spontaneous circulation (ROSC) was achieved in 60% of ICU and 70.2% of non-ICU patients.^17^ Our study also had a greater proportion of non-ICU patients (31.9%) survive in comparison to ICU (20%) but probably not statistically significant due to a small sample size.

Most of the in-hospital arrests noted were in COVID ICU possibly due to SARS-COV2 contributing to a greater severity of disease leading to arrests in the COVID unit. Thus making frequency of arrests more common in the ICU setting as opposed to wards when compared to previous studies.^1^

Mean age of patients undergoing IHCA in our study was similar in pre pandemic period, where men were affected than women^3^,^13^,^18^ but unlike a recent Finish study^19^ where sudden cardiac arrest was greater in the pre-menopausal and early postmenopausal women than younger ones, our data showed an increase in younger women getting an IHCA event. Possible causes protecting women include better hand hygiene and more likely to seek preventive care. From our study, we cannot comment if in general women access our tertiary care facility more; however, there is some evidence from primary care facilities that women access basic health units more frequently than men in Pakistan.^20^

As expected, our study also showed that lesser proportion of patients who were on mechanical ventilation achieved ROSC and STD as compared to others who were not on mechanical ventilation.^21^

4Hs and 4Ts were a common cause of poor outcomes as expected. The most common etiology of arrest in one such study was respiratory failure.^22^ Most patients during the pandemic were noted to have hypoxia making it the most common cause, because of the respiratory implications of the virus. However, since we did not note the COVID-19 status of those in Emergency, we can only presume that they were also COVID-19 positive.

### Limitations:

A limitation of our study was the small sample size as well as not having COVID-19 status of those who died in Emergency. However, despite the sample size, we were able to show some differences in pre- and during pandemic epidemiology of IHCA. A limitation is our inability to have a covid-19 status on all the patients and so some explanations were not possible.

## CONCLUSION

As expected, fewer people achieved ROSC and StD during the pandemic period in comparison to the pre-pandemic period. The role of age and gender in similar sociodemographic population needs to be further explored and tertiary facilities are encouraged to analyze and compare their IHCA outcomes pre- and during pandemic.

### Authors’ Contribution:

**FA:** Conceived, designed, collected data and prepared the manuscript.

**NG:** Analyzed data and wrote some sections of manuscript.

All authors did review, final approval of manuscript an are responsible for integrity of the study.
